# Wandering Cancer Cells: Metastatic Renal Cell Carcinoma Without Evidence of a Primary Tumor

**DOI:** 10.7759/cureus.26305

**Published:** 2022-06-24

**Authors:** Swe Swe Hlaing, Devashish Desai, Aakash Goyal, Navjot Rai, Ronald Swaab

**Affiliations:** 1 Internal Medicine, Crozer-Chester Medical Center, Upland, USA; 2 Hematology and Medical Oncology, Crozer-Chester Medical Center, Upland, USA

**Keywords:** unknown primary site, oncology imaging, renal neoplasm, renal pathology, clear renal cell carcinoma

## Abstract

Renal cell carcinoma (RCC) usually presents clinically in the advanced stage including bone metastasis. However metastatic RCC without evidence of a primary tumor in the kidney is extremely rare. We herein report a case of a 70-year-old male initially evaluated for bone lesion and diagnosed with biopsy-proven metastatic clear cell RCC without a renal primary. Given the rare nature of the disease, there is no standardized course of treatment that has yet been established. We believe that our case will add to the body of knowledge about uncommon oncologic instances and consolidate the information that has already been published.

## Introduction

The incidence of renal cell carcinoma (RCC) has been increasing in the United States [1]. It accounts for 2-3% of all solid cancers, usually occurring twice as commonly in men between the fifth and seventh decades of life. Metastasis frequently occurs in the lungs, liver, and bones [2-4]. However, bone metastasis without a primary tumor is rarely reported [5,6]. 

Given its rare occurrence, the clinical course and treatment modalities remain undefined. Multiple treatment modalities have been described in case reports, ranging from surveillance, surgical resection, chemotherapy, and/or neoadjuvant/ adjuvant radiotherapy [5,7]. However, there are limited reports of metastatic renal cell carcinoma without evidence of a primary tumor. We report a case of a 70-year-old male who presented with bone lesion, with imaging showing no primary renal tumor and biopsy of the bone mass showing clear cell carcinoma, and who is currently undergoing palliative radiation. 

## Case presentation

A 70-year-old male with a past medical history of hypertension and hyperlipidemia presented to the oncology clinic for evaluation of a bone lesion. He was initially seen in the hospital for cough and dyspnea and was diagnosed with bronchitis. CT chest performed during the visit showed incidental thoracic spine (T) 8 vertebra lesion. The following MRI of the T spine showed enhancing lesion of T8 with nonspecific findings in T7, T12, and lumbar spine (L) 1. 

**Figure 1:**
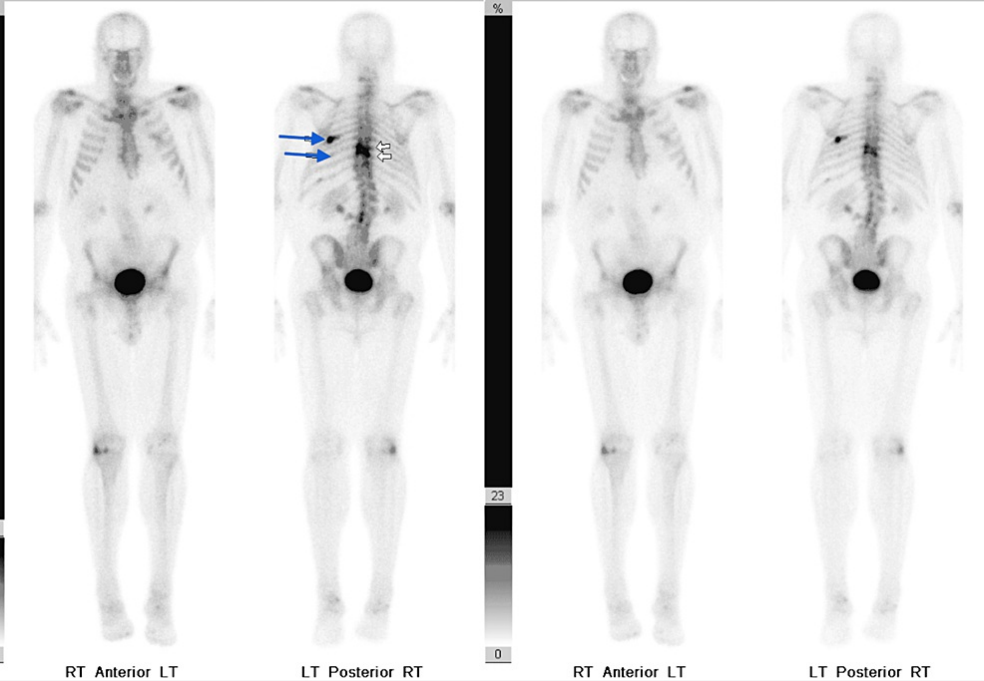
White arrows showing multifocal skeletal uptake in T1 and T8 vertebrae and blue arrows showing posterior left 6th and 10th ribs

The patient was evaluated by interventional radiology but T8 pedicle biopsy was not recommended due to its proximity to the spinal cord. The following bone scan (Figure 1) after 2 months showed multifocal skeletal uptake in T1 and T8 vertebrae and posterior left 6th and 10th ribs. The patient has been having ongoing left shoulder blade pain during this period. Serum protein electrophoresis showed an M-spike of 0.1 g/dl which was consistent with monoclonal gammopathy of undetermined significance. Prostatic specific antigen value was 4.6 and cancer antigen (CA) 19-9 was 70.

**Figure 2:**
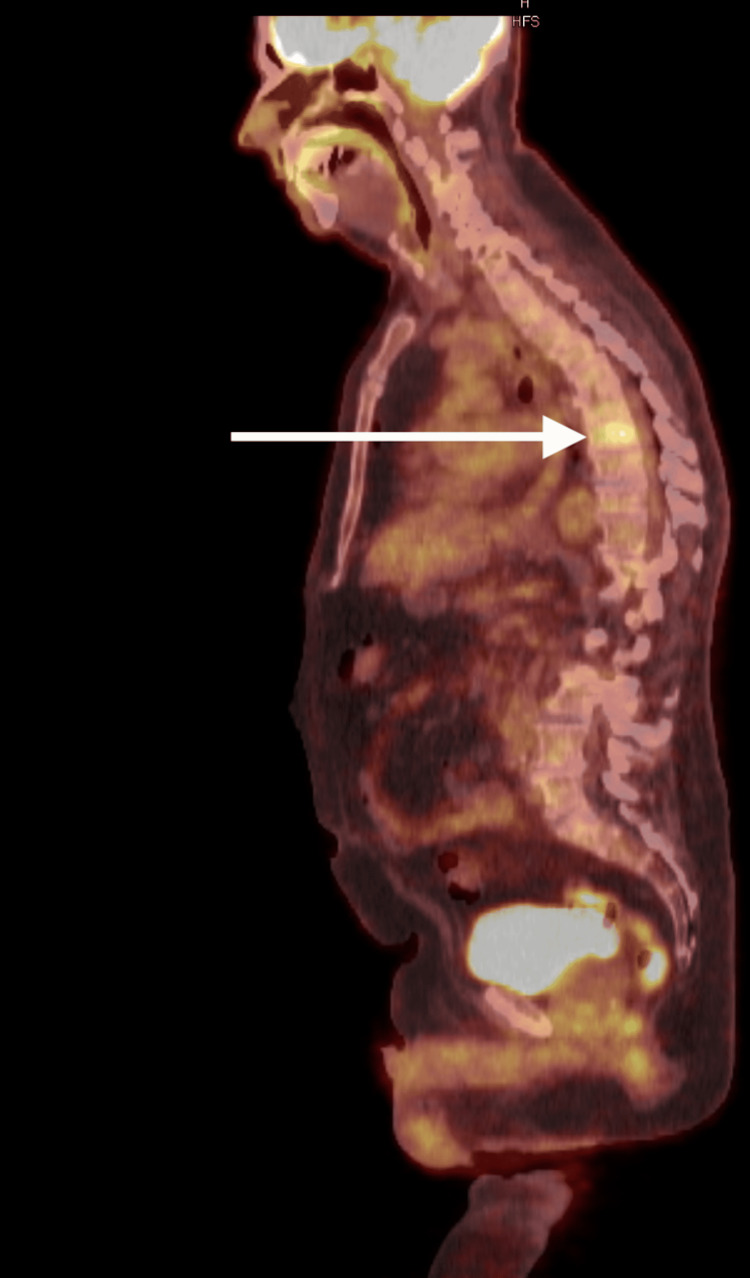
PET/CT showing osseous lytic lesion in T8 vertebra by white arrow

**Figure 3:**
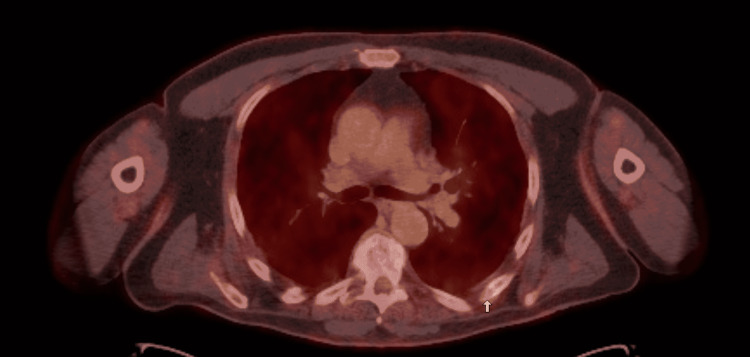
White arrow showing osseous lytic lesion in the left posterior 6th rib

The patient got a PET/CT two months after the bone scan and it showed multifocal osseous lytic lesions with mild- to moderate-range standardized update value (SUV) (Figures 2, 3). It was also noted that the largest and most intense lesions are located within the T8 vertebra and the left inferior iliac bone.

**Figure 4:**
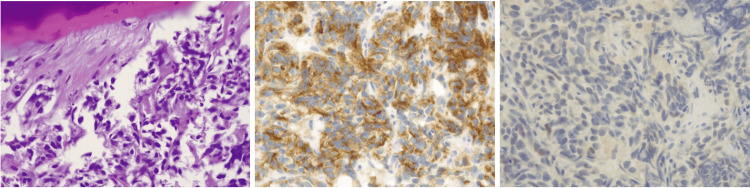
Pathology slides showing features suggestive of clear cell carcinoma Left: H&E 400x:  poorly preserved epithelial cells in nests with crush artifact, showing uniform round nuclei with indistinct chromatin pattern, indistinct cell borders, and poorly preserved cytoplasm, suggestive of clear cell features. Center: CD10 IHC 400X: positive cytoplasmic stain. Right: PAX8 IHC 400X: weak positive nuclear stain. H&E: hematoxylin and eosin stain; IHC: immunohistochemistry

Hence, the patient underwent CT guided biopsy of a lytic lesion in the left iliac bone. It showed poorly preserved epithelial cells in nests with crush artifacts showing uniform round nuclei with indistinct chromatin patterns, indistinct cell borders, and poorly preserved cytoplasm suggestive of clear cell features. A limited panel of immunohistochemical stains shows positive staining for cytokeratins (AE1/AE3), CD 10 and weak nuclear staining for PAX8, negative staining for CK7, P40, and TTF1. These results, in conjunction with the morphology in the sample, support the interpretation of metastatic clear cell renal cell carcinoma (Figure 4).

The patient was treated with palliative radiation therapy at three sites which include two posterior lateral sixth and eighth ribs, T8 and nine vertebral bodies and left upper pelvis. All sites were treated with a total dose of 30 Gy/10 fractions with the goal of pain control. He remained on surveillance. 

## Discussion

Over the years, a substantial amount of research has been done on patients with unique metastatic locations. The common sites of metastasis are lungs, liver, and bone and the exact mechanism leading to these different sites of metastasis is still unknown [8]. Understanding the diagnostic variables along with the analysis of underlying pathophysiology paves for optimization of treatment, as well as provides an approximation regarding treatment effectiveness and future prognosis. Despite the fact that there are presently no markers or diagnostic procedures that can help accurately forecast the exact time or location of metastasis, studies done over the last many years can provide a basic notion of how probable it is to return.

Stage I to III advanced RCC with primary renal origin are managed by surgical resection. However, the natural history of the disease varies widely from a few months to many years depending on the clinical, pathologic, laboratory, and radiographic features of the disease. As a result of novel medications that target the tumor vasculature or inhibit the activation of intracellular oncogenic pathways, the therapy of advanced disease has changed even more significantly [9]. Systemic therapy is typically initiated when unresectable disease, either metastatic or locally advanced, is present. 

The prognosis of metastatic RCC is generally poor [10]. A number of clinical factors for disease prognosis have been established, and these have been incorporated into the International Metastatic Renal Cell Carcinoma Database Consortium model [11]. In the modern period of therapy, this has been useful in predicting outcomes.

All these being reported, it is generally for metastatic RCC with primary tumor. There have been no established guidelines for metastatic RCC without a renal primary. In our patient, the goal of treatment was symptom control. He received palliative radiation therapy which helped him with pain control, and he remains on surveillance.

Further research and evidence-based medicine are mandatory for metastatic RCC without a renal primary. 

## Conclusions

This case is distinctive because the discovery of metastatic RCC was without kidneys being involved, as well as the only presenting symptom was bone pain. The treatment becomes very challenging as there are no established guidelines. This case highlights and adds to a growing body of literature regarding metastatic RCC without a renal primary. It also points out the importance of timely biopsy, understanding the different radiological tests, and their utility in the diagnosis of malignancy.
